# Decline of emergency admissions for cardiovascular and cerebrovascular events after the outbreak of COVID-19

**DOI:** 10.1007/s00392-020-01688-9

**Published:** 2020-08-04

**Authors:** Viktoria Schwarz, Felix Mahfoud, Lucas Lauder, Wolfgang Reith, Stefanie Behnke, Sigrun Smola, Jürgen Rissland, Thorsten Pfuhl, Bruno Scheller, Michael Böhm, Sebastian Ewen

**Affiliations:** 1 Emergency Department, Saarland University Medical Center, Homburg, Germany; 2 Clinic of Internal Medicine III (Cardiology, Angiology and Intensive Care Medicine), Saarland University Medical Center, Universitätsklinikum des Saarlandes, Saarland University, Kirrberger Str. 100, Geb. 41, 66421 Homburg, Saar Germany; 3 Department of Neuroradiology, Saarland University Medical Center, Homburg, Germany; 4 Institute of Virology, Saarland University Medical Center, Homburg, Germany; 5 Department of Neurology, Saarland University Medical Center, Homburg, Germany

**Keywords:** COVID-19, SARS-CoV-2, Acute coronary syndrome, Cardiovascular events, Cerebrovascular events

## Abstract

**Background:**

The spread of the novel coronavirus SARS-CoV-2 and the guidance from authorities for social distancing and media reporting lead to significant uncertainty in Germany. Concerns have been expressed regarding the underdiagnosing of harmful diseases. We explored the rates of emergency presentations for acute coronary syndrome (ACS) and acute cerebrovascular events (ACVE) before and after spread of SARS-CoV-2.

**Methods:**

We analyzed all-cause visits at a tertiary university emergency department and admissions for ACS and ACVE before (calendar weeks 1–9, 2020) and after (calendar weeks 10–16, 2020) the first coronavirus disease (COVID-19) case in the region of the Saarland, Germany. The data were compared with the same period of the previous year.

**Results:**

In 2020 an average of 346 patients per week presented at the emergency department whereas in 2019 an average of 400 patients presented up to calendar week 16 (*p* = 0.018; whole year 2019 = 395 patients per week). After the first COVID-19 diagnosis in the region, emergency department visit volume decreased by 30% compared with the same period in 2019 (*p* = 0.0012). Admissions due to ACS decreased by 41% (*p* = 0.0023 for all; Δ − 71% (*p* = 0.007) for unstable angina, Δ − 25% (*p* = 0.42) for myocardial infarction with ST-elevation and Δ − 17% (*p* = 0.28) without ST-elevation) compared with the same period in 2019 and decreased from 142 patients in calendar weeks 1–9 to 62 patients in calendar weeks 10–16. ACVE decreased numerically by 20% [*p* = 0.25 for all; transient ischemic attack: Δ − 32% (*p* = 0.18), ischemic stroke: Δ − 23% (*p* = 0.48), intracerebral haemorrhage: Δ + 57% (*p* = 0.4)]. There was no significant change in ACVE per week (*p* = 0.7) comparing calendar weeks 1–9 (213 patients) and weeks 10–16 (147 patients). Testing of 3756 samples was performed to detect 58 SARS-CoV-2 positive patients (prevalence 1,54%, thereof one patient with myocardial and two with cerebral ischemia) up to calendar week 16 in 2020.

**Conclusions:**

The COVID-19 pandemic was associated with a significant decrease in all-cause admission and admissions due to cardiovascular events in the emergency department. Regarding acute cerebrovascular events there was a numerical decrease but no significant difference.

## Introduction

The ongoing pandemic of the novel coronavirus SARS-CoV-2 disease (COVID-19) unsettles people worldwide and has led to 287,399 deaths globally by May 13, 2020 [1]. To protect the local health system from overload and to maintain intensive care capacities for COVID-19 patients, elective admissions have been reduced and postponed. In addition to hygiene advices, the World Health Organization (WHO) strongly recommends limiting face-to-face contact with others via social distancing [2]. Subsequently, changes in the pattern of hospital admissions have been observed [3–6]. Valuable time passes with the ubiquitous differential diagnosis of respiratory insufficiency caused by COVID-19, which is frequently aimed to exclude before detailed examinations (e.g., physical examination, ultrasound, computed tomography, coronary angiography) take place [7]. Therefore, concerns have been raised regarding underdiagnosing of harmful but treatable cardiovascular diseases.

## Methods

### Study design and patients

We analyzed ICD-10-GM (International Statistical Classification of Diseases and Related Health Problems, 10th Revision, German Modification) codes from discharge letters at Saarland University Medical Center, Germany, during calendar weeks 1–16 of the years 2019 and 2020, respectively. We assessed the all-cause visit volume at the emergency department of Internal Medicine (aged ≥ 18 years) and Surgery (aged ≥ 0 years). Digital records of patients admitted for acute coronary syndrome (ACS), including unstable angina pectoris as well as myocardial infarction with (STEMI) or without (NSTEMI) ST-elevation were evaluated. Similarly, the number of acute cerebrovascular events (ACVE), including transient ischemic attack, ischemic stroke, and intracerebral haemorrhage were evaluated. We compared calendar weeks 1–9 before the first COVID-19 case in the region of Saarland (1st case on March 3rd, calendar week 10) with calendar weeks 10–16, during which the infection numbers in the region increased. Focus was also given on the timing of the shutdown in the Saarland (March 16th, calendar week 12). To reduce bias due to seasonal effects, we compared the visit volume with the same period of the previous year.

### RT-PCR-based diagnostic of COVID-19

The indication for COVID-19 testing was performed according to the recommendations of the Robert Koch Institute [8]. COVID-19 confirmation test was performed by isolated RNA from eSwabs (Copan Italia, Brescia, Italy) using the NucliSens easy MAG Instrument (bioMeriéux Deutschland, Nürtingen, Germany) following the manufacturers’ instructions. PCR amplification used the RealStar SARS-CoV-2 RT-PCR Kit 1.0 RUO (Altona Diagnostics, Hamburg, Germany) on a Light Cycler 480 II Real-Time PCR Instrument (Roche Diagnostics Deutschland, Mannheim, Germany) according to the manufacturers’ instructions [9]. From week 12 on COVID-19 confirmation was additionally performed with the cobas^®^ SARS-CoV-2 Test for the cobas^®^ 6800 instrument (Roche Diagnostics GmbH, Mannheim, Germany) according to manufacturers’ instructions. Only tests from patients at the Saarland University Medical Center were included in the analyses, tests from staff were excluded.

### Statistics

Data are presented as mean unless otherwise specified. Categorical variables are presented as numbers (%). Variables were tested for normality utilizing the Shapiro–Wilk test. For continuous variables, between-group comparisons were performed using the Mann–Whitney or Wilcoxon rank-sum test and Kruskal–Wallis-test where appropriate. Significance tests were two-tailed with *p* < 0.05 considered significant. All statistical analyses were performed using GraphPad Prism version 8.2.1 (GraphPad Software, La Jolla, CA, USA).

## Results

### Emergency department visit volume

**Fig. 1:**
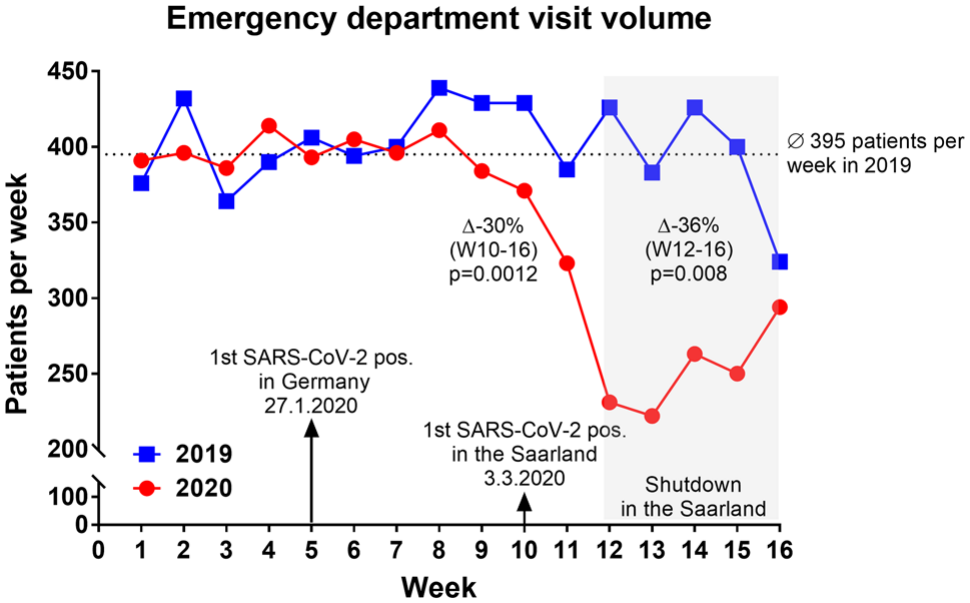
Emergency department visit volume. Comparison of the number of patients during calendar weeks 1–16 in 2019 and 2020

**Fig. 2:**
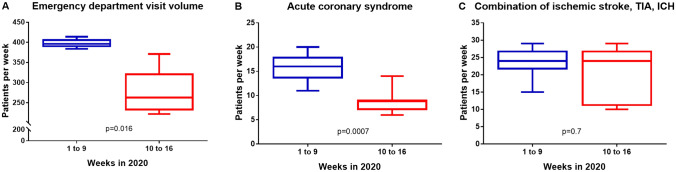
Box plot of patients at calendar weeks 1–9 and 10–16 in the year 2020. **a** Emergency department visit volume. **b** Acute coronary syndrome. **c** Combination of ischemic stroke, transient ischemic attack (TIA) and intracerebral haemorrhage (ICH)

At the emergency department in 2020 an average of 346 patients per week presented up to calendar week 16 whereas in 2019 an average of 400 patients per week presented (*p* = 0.018) over the same period of time. In 2020, admissions at the emergency department decreased by calendar week 8 parallel to the occasional detection of COVID-19 in Germany (Fig. 1). After verification of the first patient with COVID-19 in the Saarland region on March 3, 2020 (calendar week 10), the number of admissions dropped, regardless of the reason for admission (average of 397 patients per week in calendar weeks 1–9 *vs.* average of 279 patients per week in calendar weeks 10–16, *p* = 0.016) (Fig. 2a).There was no significant difference for the average visit volume when comparing weeks 1–9 of 2020 with 2019. After the first COVID-19 diagnosis in the region, all-cause emergency department visit volume decreased by 30% compared with the same period in 2019 (1954 patients in 2020 *vs.* 2773 patients in 2019 at calendar weeks 10–16, *p* = 0.0012) and tended to further decrease by 36% after the shutdown in the Saarland compared with the same period in 2019 (1260 patients in 2020 *vs.* 1959 patients in 2019 at calendar weeks 12–16, *p* = 0.008). In parallel to the regional shutdown, the nadir was reached at calendar weeks 12–13. Following calendar week 14, the visit volume at the emergency department increased again but remained significantly lower compared with the previous year.

### Acute coronary syndrome in the emergency department

**Fig. 3:**
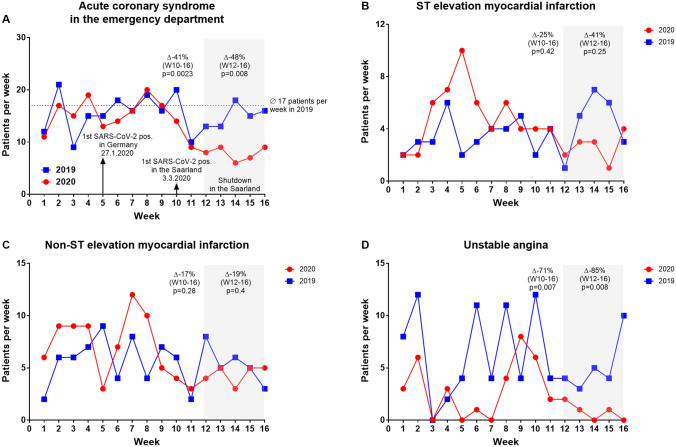
Acute coronary syndrome. **a** Accumulated number of patients with acute coronary syndrome in the emergency department at calendar weeks 1–16 in the year 2019 and 2020. Number of patients at calendar weeks 1–16 in the year 2019 and 2020 with myocardial infarction with (**b**, STEMI) and without (**c**, NSTEMI) ST-elevation and unstable angina (**d**)

After the first laboratory-confirmed SARS-CoV-2 infection in calendar week 10 of 2020 in the Saarland, the number of patients diagnosed with an ACS decreased significantly by 41% compared with the previous year (62 patients in 2020 *vs.* 105 patients in 2019 at calendar weeks 10–16, *p* = 0.0023) (Fig. 3). During the shutdown in calendar weeks 12–16, there was a further reduction of patients who admitted with ACS compared with the previous year (Δ − 48%, 39 patients in 2020 *vs.* 75 patients in 2019 at calendar weeks 12–16, *p* = 0.008). There was no significant difference in admission rates when comparing week 1–9 of the years 2020 to 2019. Regarding the different entities of ACS, we observed a significant decrease in unstable angina by 71% after the first confirmed COVID-19 patient in the region (12 patients in 2020 *vs.* 42 patients in 2019, *p* = 0.007) respectively a decrease by 85% during the lockdown (4 patients in 2020 *vs.* 27 patients in 2019, *p* = 0.008) compared with the previous year. The decline in STEMI (Δ − 25% in calendar weeks 10–16, 21 patients in 2020 *vs.* 28 patients in 2019, *p* = 0,42; Δ − 41% in calendar weeks 12–16, 13 patients in 2020 *vs.* 22 patients in 2019, *p* = 0.25) and NSTEMI (Δ − 17% in calendar weeks 10–16, 29 patients in 2020 *vs.* 35 patients in 2019, *p* = 0.28; Δ − 19% in calendar weeks 12–16, 22 patients in 2020 *vs.* 29 patients in 2019, *p* = 0.4) was not significant. However, in weeks 1–9 more ACS were treated compared with period after the first confirmed COVID-19 case in the region (142 patients in calendar weeks 1–9 *vs.* 62 patients in calendar weeks 10–16, *p* = 0.0007) (Fig. 2b).

### Acute cerebrovascular events

**Fig. 4:**
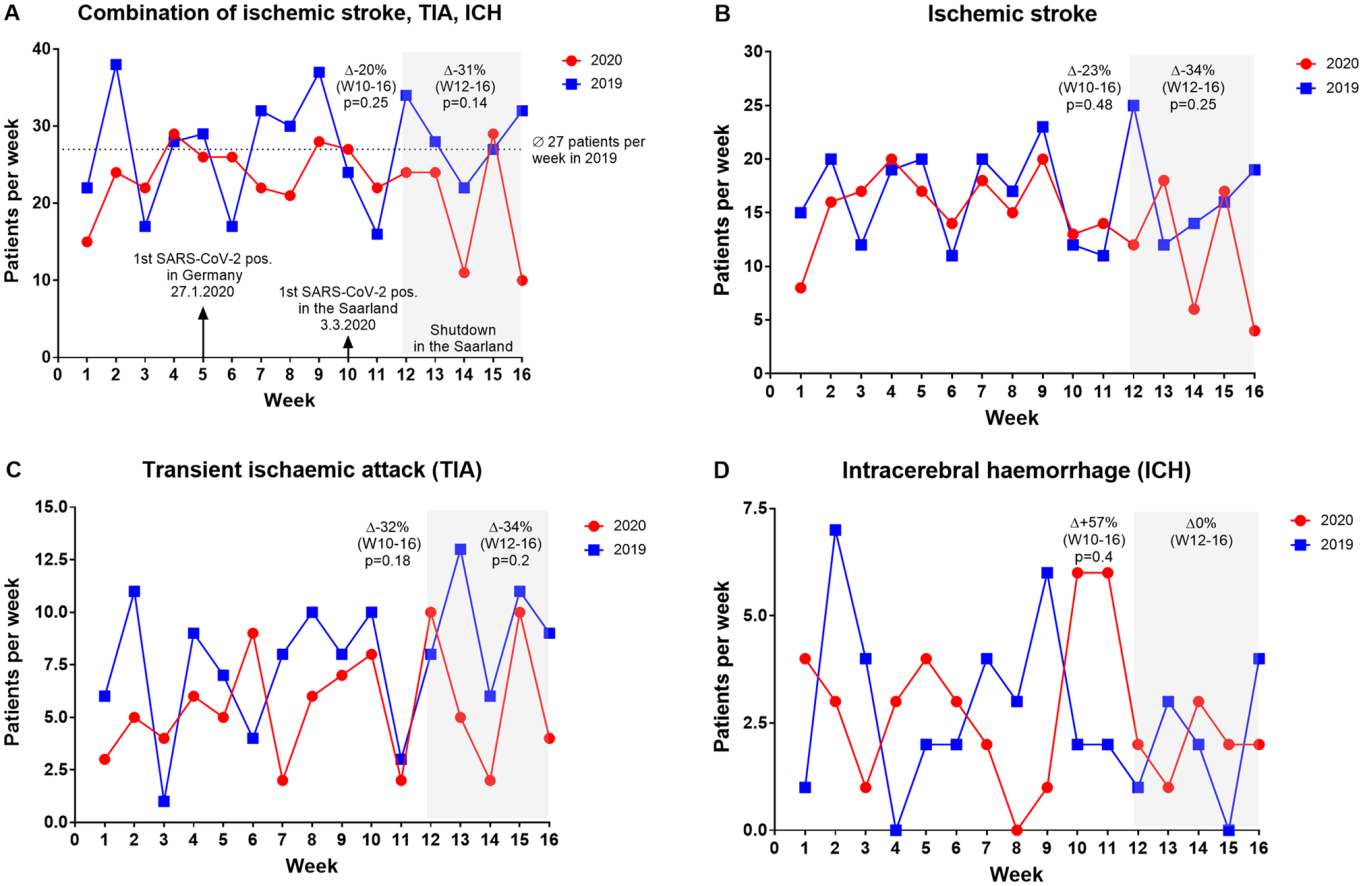
Acute cerebrovascular events. **a** Accumulated number of patients with ischemic stroke, transient ischemic attack (TIA) and intracerebral haemorrhage (ICH) at calendar weeks 1–16 in the year 2019 and 2020. Number of patients at calendar weeks 1–16 in the year 2019 and 2020 with ischemic stroke (**b**), TIA (**c**) and ICH (**d**)

Admissions due to ACVE, including transient ischemic attack, ischemic stroke, and intracerebral haemorrhage were numerically reduced by 20% at calendar week 10 (147 patients in 2020 *vs.* 183 patients in 2019, *p* = 0.25) and by 31% during shutdown (98 patients in 2020 *vs.* 143 patients in 2019, *p* = 0.14) compared with the same period last year (Fig. 4). Looking at the individual entities at calendar week 10–16 and under lockdown conditions compared to the same period in 2019 there was a non-significant reduction by 32% (41 patients in 2020 *vs.* 60 patients in 2019, *p* = 0.18) and 34% (31 patients in 2020 *vs.* 47 patients in 2019, *p* = 0.2) of transient ischemic attack, a 23% (84 patients in 2020 *vs.* 109 patients in 2019, *p* = 0.48) and 34% (57 patients in 2020 *vs.* 86 patients in 2019, *p* = 0.25) non-significant reduction of ischemic strokes as well as a 57% (22 patients in 2020 *vs.* 14 patients in 2019, *p* = 0.4) and 0% (10 patients in 2020 *vs.* 10 patients in 2019, *p* = 1) also not significant increase in intracerebral haemorrhage. There was no significant difference when comparing the mean number of presentations per week for acute cerebral events at week 1–9 and 10–16 of the year 2020 (24 patients per week in 2020 *vs.* 8 patients per week in 2019, *p* = 0.7) (Fig. 2c).

### Virological test volume for COVID-19

**Fig. 5:**
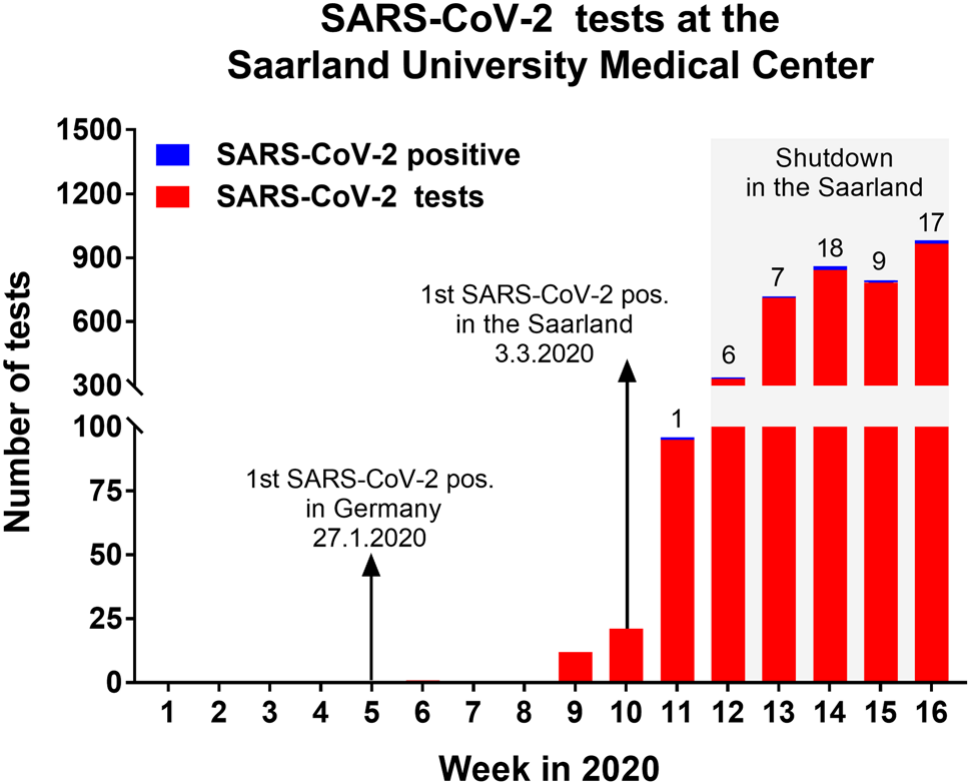
Virological test volume for SARS-CoV-2 for patients at the Saarland University Medical Center

With spread of the infection the test volume for SARS-CoV-2 testing increased week to week (one test in calendar week 6, 965 tests in calendar week 16; Fig. 5). In our analyses during end of week 16 in 2020, testing of 3765 samples detected 58 positive patients. The overall rate of positive tests from patients of the Saarland University Medical Center up to calendar week 16 was 1.54%. We identified one patient with acute myocardial infarction and one patient with ACVE with coincidental SARS-CoV-2 infection. One patient with previously known COVID-19 infection was admitted with an acute cerebrovascular event.

## Discussion

At the Saarland University hospital, a tertiary referral center, a significant decrease in medical contacts at the emergency department and the number of patients presenting with ACS was documented during the first wave of the COVID-19 pandemic. This was mainly driven due to an 85% decrease in admission for unstable angina during shutdown conditions whereas the reduction in admissions caused by myocardial infarction was not significant. Regarding acute cerebrovascular events there was a numerically reduction in our analyses.

During the past weeks, reports on increasing numbers of COVID-19 positive patients in China, US and Europe dominated the reporting in the lay press and resulted in pressure on health care systems like in Northern Italy. This coverage led to great fear and uncertainty in several countries, including Germany [10]. In England attendances at the emergency department have fallen by 25% the week after the prime minister Boris Johnson announced a lockdown on 23 March [11]. Similar decline of emergency department visits could also be observed for paediatric emergency department visits [6]. To exclude a seasonal effect in the analyses, we compared the visit volume with the same period of the previous year. Proportionally more patients who had been admitted to the emergency department subsequently required inpatient treatment, indicating that consultations for less severe reasons decreased. In calendar weeks 15–16, the emergency department visit volume graphs of 2019 and 2020 get closer, which may have several reasons like behavior and travelling attitudes. Exemplarily, Christmas and Midsummer holidays have been associated with higher risk of myocardial infarction; a weekend-effect with delay in coronary angiography for NSTEMI resulting in a higher mortality has been described [12, 13]. Behavior during the Easter holidays may have been different in 2020 due to the government restrictions in Germany. In 2019 (Easter at calendar week 16), the festivity with the family and/or a short vacation was obvious, so that more trivial health problems potentially were solved in the family itself and a relevant number of people were located abroad. In 2020 (Easter at calendar week 15) neither a family visit nor a journey was allowed due to the lockdown in our region. Patients who had previously postponed their doctor’s appointment may have gone to see a doctor due to the increasing level of suffering.

We found an “atypical” low number of cardiovascular events compared with the weeks before the regional outbreak of the pandemic and compared with the same period of the previous year. A similar trend was observed in cardiovascular centers in Austria [14], the severely affected Northern Italy [3] and Iran [15]. It can be assumed by a stable incidence of patients having an ACS that patients abstain from seeing a doctor despite symptoms of acute myocardial ischemia. This is in line with the increasing number of out-of-hospital cardiac arrest in Italy compared with the same period in 2019 [4]. In COVID-19 patients, cardiac injury is a common feature whereby the mechanisms have not yet been finally clarified [16]. In our analyses, there was only one patient with myocardial infarction who was tested positive for the virus, but regarding a total number of 62 patients presenting with ACS the ratio was 1,6%—and is therefore in the area of the overall prevalence in our hospital. A significant reduction in catheterization laboratory activations during COVID-19 pandemic due to a decline in patients with STEMI was observed in the US [17].

Although the difference was not statistically significant, we documented a numerically lower number of transient ischemic attacks and ischemic strokes. According to the World Stroke Organization there is a significant fall in stroke presentations globally [5]. One may speculate that patients with mild or moderate symptoms refrain from consulting health care providers. The increase in intracerebral haemorrhage could be (1) directly attributed to underdiagnosed and therefore undertreated cerebral ischemia, (2) less strictly controlled blood pressure values due to reduced contacts with doctors or (3) an increase of accidents in the household (e.g., due to self-made construction work or family violence during the shutdown and government restrictions) [18–21].

Coagulopathy, inflammatory responses, and endothelial dysfunction are associated with COVID-19 [22–24]. As a result of this, with (symptomatic as well as asymptomatic) spread of SARS-CoV-2 infection an increased rate of acute cardiovascular and acute cerebrovascular events can be assumed [25]. The reduction in ED numbers was not substantiated by an objective increase of COVID-19 infections. Despite testing the day of admission, by now, we identified only one patient with acute myocardial infarction and one patient with acute cerebrovascular event with coincidental SARS-CoV-2 infection. In addition, only one patient with previously known COVID-19 was admitted with an acute cerebrovascular event. Given the overall low number of positive test at our hospital, patients’ concern about an increased risk of infection in the hospital appears unsubstantiated.

As a result of the legal provisions that no non-urgent or preventive medical examinations should take place during the shutdown, it can be assumed that a high rate of underdiagnosed diseases can be expected. The general recommendation to reduce social contacts in everyday life wherever and whenever possible led more people to stay at their presumed safe homes. This recommendation could have impaired respectively delayed scheduled and unscheduled doctor visits. Whether the rate of successfully treated COVID-19-positive patients outweighs the high number of undiagnosed diseases caused by the shutdown remains to be answered. It would be fatal if there would be an increase in deaths—especially in those without COVID-19 having harmful, but treatable diseases.

## Limitations

Patients presented directly at the department of neurology and pediatrics or transferred directly to specialized departments were not included in the analysis of the total number of patients presented at the central emergency department at the Saarland University Medical Center.

## Conclusions

The COVID-19 pandemic is associated with a significant decrease in all-cause admission and admissions due to acute cardiovascular and potentially cerebrovascular events in the emergency department. Regarding acute cerebrovascular events there was a numerical difference which needs to be scrutinized in large sample series. Increase of test capacity adapted to the pandemic process up to the general testing for inpatients has been succeeded in order to be able to maintain standard medical care and offers chances to restart indicated preventive medicine. Essential part of successful medical treatment maintains the step that the patient comes to see a doctor at all. As a result of these observations, regional media campaigns as already started are important to encourage patients to continue to see a doctor if they have health-related problems.

## Data Availability

All data is available and can be provided if requested.
